# Supplementary material for: Real-World Data of First 12-Months of Ofatumumab Treatment in Multiple Sclerosis Patients—A Multicenter Experience from Tertiary Referral Centers
Source: Medicina (Kaunas). 2025 Aug 31;61(9):1568. doi: 10.3390/medicina61091568 (PMC12472045; doi:10.3390/medicina61091568)
Supplement: Supplementary file 1 [file medicina-61-01568-s001.zip › medicina-3833328-supplementary.pdf]

Supplementary Material

Supplementary Table 1. Proportions and 95% confidence intervals (95% CI) for all point estimates.

Methods note. For all proportions, 95% confidence intervals were calculated using the Wilson score method.

A) Clinical and radiological efficacy outcomes at 12 months

Outcome	Overall (n=142) n/N (%)	95% CI	Switch (n=95) n/N (%)	95% CI	Naïve (n=47) n/N (%)	95% CI
Relapses	17/142 (12.0%)	7.6–18.3%	12/95 (12.6%)	7.4–20.8%	5/47 (10.6%)	4.6–22.6%
New/enlarged T2-w lesions	18/142 (12.7%)	8.2–19.1%	12/95 (12.6%)	7.4–20.8%	6/47 (12.8%)	6.0–25.2%
Gadolinium-enhancing lesions (GELs)	5/142 (3.5%)	1.5–8.0%	3/95 (3.2%)	1.1–8.9%	2/47 (4.3%)	1.2–14.2%
EDSS progression	18/142 (12.7%)	8.2–19.1%	13/95 (13.7%)	8.2–22.0%	5/47 (10.6%)	4.6–22.6%
EDSS improvement	20/142 (14.1%)	9.3–20.8%	12/95 (12.6%)	7.4–20.8%	8/47 (17.0%)	8.9–30.1%
NEDA-3	108/142 (76.1%)	68.4–82.3%	72/95 (75.8%)	66.3–83.3%	36/47 (76.6%)	62.8–86.4%

B) Safety outcomes at 12 months

Outcome	Overall (n=184) n/N (%)	95% CI	Switch (n=95) n/N (%)	95% CI	Naïve (n=47) n/N (%)	95% CI
Any adverse event (AE)	123/184 (66.8%)	59.8–73.2%	62/95 (65.3%)	55.3–74.1%	34/47 (72.3%)	58.2–83.1%
Injection-related reactions (IRR)	116/184 (63.0%)	55.9–69.7%	57/95 (60.0%)	49.9–69.3%	31/47 (66.0%)	51.7–77.8%
Infections (any grade)	30/184 (16.3%)	11.7–22.3%	18/95 (18.9%)	12.3–28.0%	7/47 (14.9%)	7.4–27.7%

Abbreviations: EDSS, Expanded Disability Status Scale; T2-w, T2-weighted; GELs, gadolinium-enhancing lesions; NEDA-3, no evidence of disease activity-3; AE, adverse event; IRR, injection-related reaction.
